# Robotic-Assisted Surgery for Cadaveric Skull Opening: A New Method of Autopsy Procedure

**DOI:** 10.3389/frobt.2020.622083

**Published:** 2021-02-17

**Authors:** Arnon Jumlongkul, Panuwat Chutivongse

**Affiliations:** ^1^ School of Medicine, Mae Fah Luang University, Chiang Rai, Thailand; ^2^ Department of Forensic Medicine, Faculty of Medicine, Chulalongkorn University, Bangkok, Thailand

**Keywords:** aerosol, autopsy noise, bone dust, oscillating saw, robotic-assisted surgery, robotic autopsy saw

## Abstract

**Background:** Sawing of bone is an essential part of an autopsy procedure. An oscillating saw always generates noise, fine infectious dust particles, and the possibility of traumatic injuries, all of which can induce occupational hazard risks to autopsy workers, especially during the COVID-19 pandemic.

**Objectives:** The first goal of this study was to explore the production of noise and bone dust emission, comparing an oscillating saw and a robotic autopsy saw during an autopsy. The second goal was to evaluate the performance of a new robotic autopsy method, used during skull opening. The third goal was to encourage mortuary workers to use robotic technology during the autopsy procedure to protect us away from occupational injuries as well as airborne infections.

**Materials and Methods:** The experiments involved a comparison of noise levels and aerosol production during skull cutting between the oscillating saw and the robotic autopsy saw.

**Results:** The results confirmed that noise production from the robotic autopsy saw was lower than the oscillating saw. However, the bone dust levels, produced by the robotic autopsy saw, were greater than the oscillating saw, but were not greater than the dust concentrations which were present before opening the skull.

**Conclusions:** The use of a new robotic system might be an alternative choice for protecting against occupational damage among the healthcare workers. Further research might attempt to consider other healthcare problems which occur in the autopsy workplace and apply the robotic-assisted technology in autopsy surgery.

## Introduction

‘Autopsy’ is a surgical procedure performed by pathologists, the purpose of which is to discover the cause of death, the manner of death, the mechanism of death, and any other issues related to the death. The autopsy must be done even though the COVID-19 situation has been going on. There are two types of autopsies, namely a medico-legal or forensic autopsy (i.e., autopsy, which is performed for legal purposes) and a clinical or academic autopsy (i.e., autopsy that is requested by physicians for reasons other than those required for legal purposes). An autopsy includes the external examination of the body and dissection of internal organs from many sites of body cavities, namely cranial, thoracic, abdominal, and pelvic cavities (Ayoub and Chow, 2008; Pluim et al., 2018). To make the external cranial examination, the pathologists usually inspect and clarify the features of the cadaver’s head, identify any external wounds, take photographs, and incise the scalp across the posterior vertex from behind the ears. The tissues are reflected in the lower forehead and occiput for preparing the bone cut. The skull is sawn by hand or power tools and the calvarium is then removed after this cut is completed. To start the intracranial examination, the dura is incised to allow brain removal, and the base of the skull is examined. The brain is brought into a pan for measuring and weighing before further dissection and tissue fixation (Pekka Saukko, 2015).

The human skull was originally cut using a hand saw, which consumed considerable time and labor. Occasionally, a slippery skull could not be held firmly, causing accidental operator injuries. Therefore, many types of electric autopsy saws were developed for this work, including both band and circular saws, but these are prone to causing hand injuries. Oscillating saws, which are routinely used during autopsy operation, always generate aerosol production from bone cutting and so can spread hazardous pathogens, such as Human Immunodeficiency Virus (HIV), Hepatitis B and C, Streptococci, etc. In post-mortem COVID-19 cases, SARS-CoV-2 and its viral protein could be found in the brains and cranial nerves, respectively (Matschke et al., 2020). Sometimes airborne infections, especially tuberculosis, can involve the human skull, also called ‘calvarial tuberculosis’(Rosli and Harun, 2016). Saw blade frequency and saw blade contact loads on the bone affect the production of these aerosols. However, almost all forensic autopsies have operated for either unnatural or sudden death cases that we also do not know their COVID-19 exposed histories. Consequently, mortuary staff may expose any hazardous micro-organisms inevitably.

Vast amounts of aerosol particles can be produced by the oscillating saw, measuring 0.3–10 μm, while the bone band saw generates respirable aerosolized particles between 0.3 and 5 µm in diameter (Wenner et al., 2017; Pluim et al., 2018). For human safety, oscillating saws are used for skull cutting, but still produce heavy noise pollution and bone dust. The modified type of ‘oscillating saw with spray-tube’ and ‘oscillating saw with exhauster’ were invented to help prevent co-workers exposure to airborne infection (Kernbach-Wighton et al., 1996; Kernbach-Wighton et al., 1998). Additionally the noise hazard in an operating room can adversely affect the inner ear structure and can cause noise-induce hearing loss (NIHL) among surgeons and their colleagues. The peak sound level in an operating theater may exceed 140 dBA. To reduce the intensity of the noise from bone surgery, the use of oscillating tip saw systems are preferred to oscillating saw blade systems (Peters et al., 2016; Razali et al., 2017).

According to the development of robotic-assisted surgery, previous studies showed some advanced surgical technologies, for instance, firstly, the use of an improved recurrent neural network (RNN) scheme for controlling the trajectory of redundant robot manipulators, which has been used for the surgical tasks related to tumor resection skills (Su et al., 2020a). Secondly, the teleoperated Minimally Invasive Surgery (MIS) using an improved human-robot collaborative control (IHRCC) scheme, this technique can improve the accuracy of both a remote center of motion (RCM) constraint and also surgical tip (Su et al., 2019). Thirdly, the incorporation of an Internet of Things and Robot-assisted Minimally Invasive Surgery, which can improve the RCM constraint as well as surgical tip (Su et al., 2020b). Although massive articles have focused on the improvement of surgical technique within a human, unfortunately, medical personnel and also engineers scarcely think about how to apply human-robot interaction for mortuary tasks.

Nowadays the most commonly used autopsy saw is an oscillating saw, without spray-tube or suction, and the workers are therefore still exposed to the risk of injuries, pathogens from bone dust and secretions, and loud noise pollution. Therefore, the first goal of this study was to explore the production of noise and bone dust emission between a traditional oscillating saw and a robotic autopsy saw, which the authors just made before, when used in an autopsy room during the autopsy. The second goal was to evaluate the performance of robotic autopsy saw which represents a new method of skull opening. Finally, to encourage mortuary workers to use robotic technology to protect us away from occupational injuries as well as airborne infections, especially tuberculosis and also COVID-19.

## Materials and Methods

According to the lack of robotic study in the field of forensic medicine, then, this article was a pilot and an experimental study. The tests of the 2-machine also included average noise levels and also the number of aerosol particles. Experimental details are shown below.

### Subjects

Cadaveric subjects, who died from May 2018 to January 2019 as a result of unnatural deaths, were brought to the Department of Forensic Medicine, Faculty of Medicine, Chulalongkorn University, Bangkok, Thailand for a forensic autopsy. The inclusion criteria were as follows:

1. The cadavers were adult 20–70 years old;
2. There was no history of osteoporosis or disease related to malformation of bone.

### The exclusion criteria also included

1. Either the scalp or skull presented pathologic or traumatic findings when inspected by gross examination.

Ten corpses were selected for this research. Eight deceased subjects were male (age 23–57 years, mean age 42.6 years) and two cadavers were female (age 56 and 65 years, mean age 60.5 years). The complete forensic autopsies were performed at the Chulalongkorn Forensic Medicine Center, Department of Forensic Medicine, Faculty of Medicine, King Chulalongkorn Memorial Hospital. This research was permitted by the Institutional Review Board of the Faculty of Medicine, Chulalongkorn University, Bangkok, Thailand (COA No. 880/2016, IRB No. 557/59). Written informed consent was obtained from the corpse’s legal guardian/next of kin for the publication of any potentially identifiable images or data included in this article.

### Experimental Setup

**FIGURE 1:**
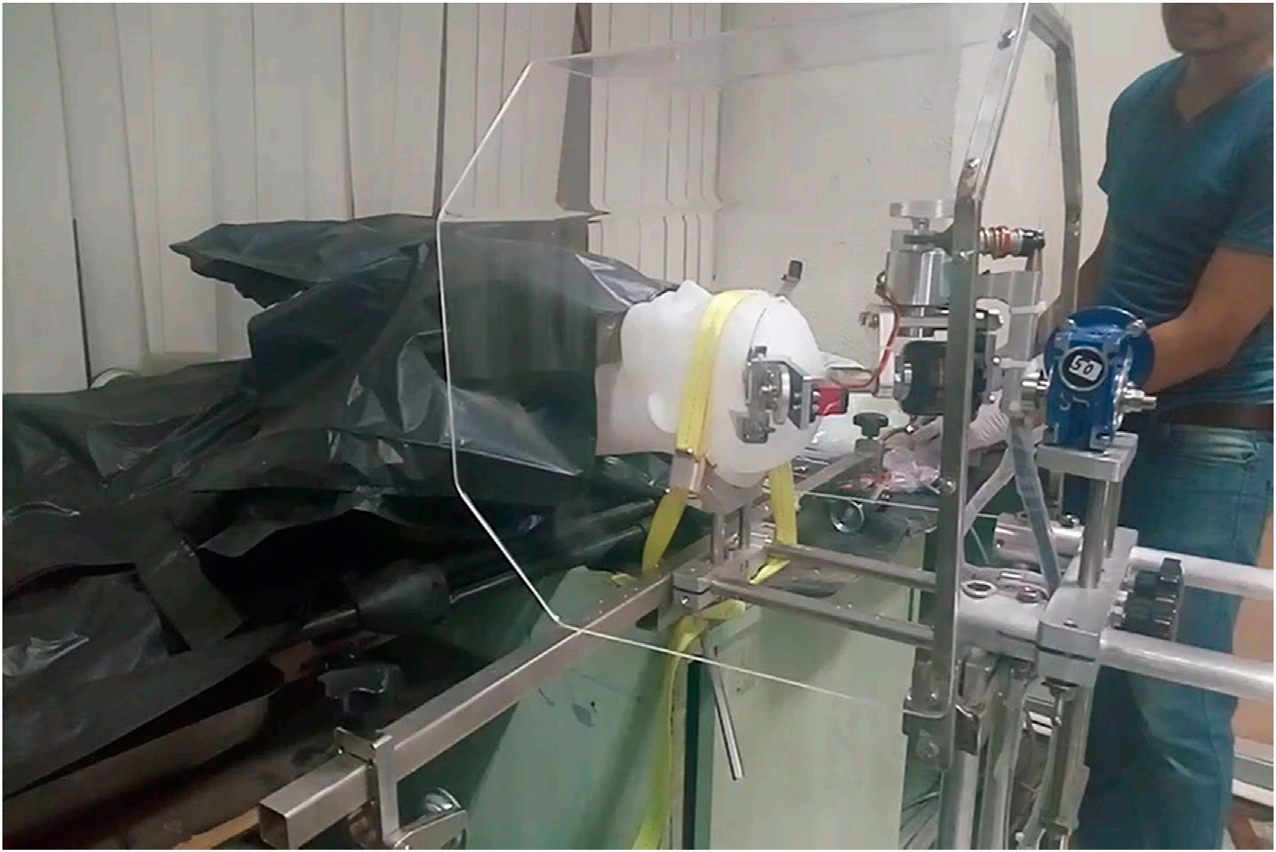
The plastic head model, which in place of the human skull, was set in a supine position.

The cadavers were divided into two groups; five cadavers for oscillating saw testing and five cadavers for robotic autopsy saw testing. The deceased subjects were positioned in a supine position. Scalp opening was done. There was no autopsy procedure before, during, and after each experiment for at least 30 min. All experiments were performed on the same autopsy bed which was 80 cm high. Both the sound level meter and real-time dust monitor were placed at the same reference point, at a distance of 100 cm away from the calvarium on the horizontal axis and parallel to the calvarium. All skull cutting procedures, except the forensic physicians, were done by the same mortuary team. The position of cadaver is shown in Figure 1.

### Autopsy Saw Instruments

#### Oscillating Saw

An oscillating autopsy saw (SG-700–01, SCHREIBER GmbH), 220–240 V 50 Hz 250 W strokes 12,000–21,000/min with segment saw blade (model SG-400–08), was used for this research. The sawing rotation and the saw blade depth were controlled at the surgical site (SCHREIBER GmbH, 2012).

#### Robotic Autopsy Saw

**FIGURE 2:**
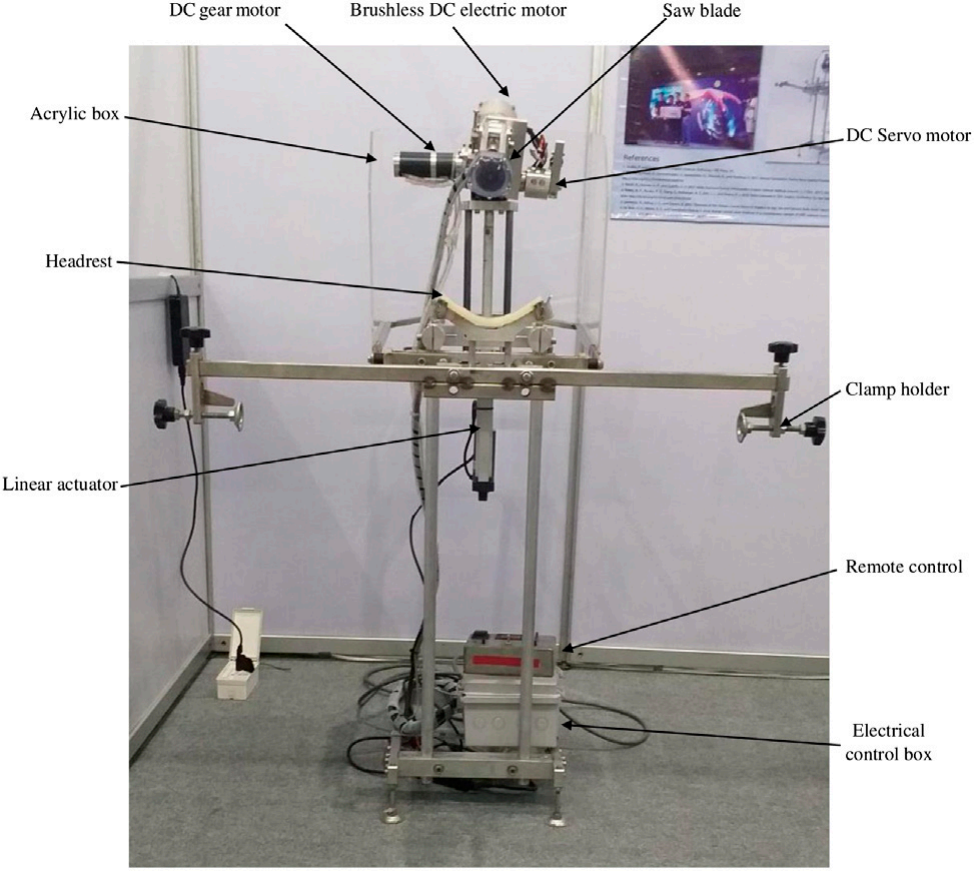
The entire part of the robotic autopsy saw.

**FIGURE 3:**
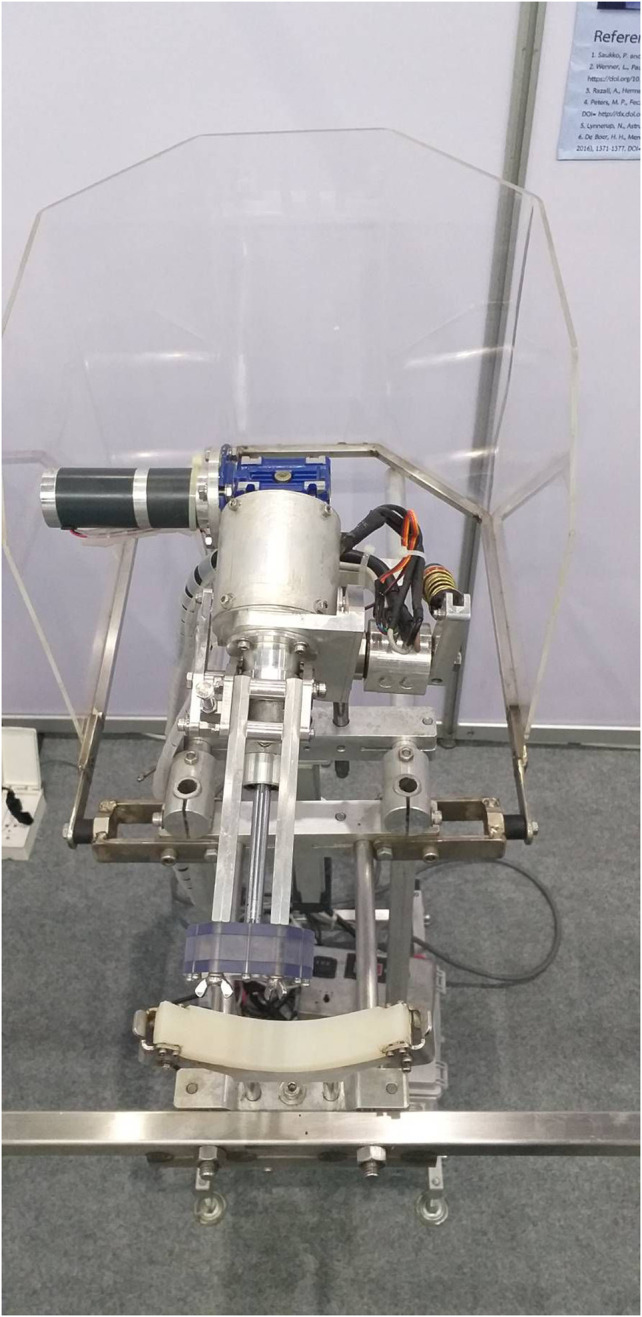
The saw frame set of the robotic autopsy saw which can turn around the cadaveric skull.

**FIGURE 4:**
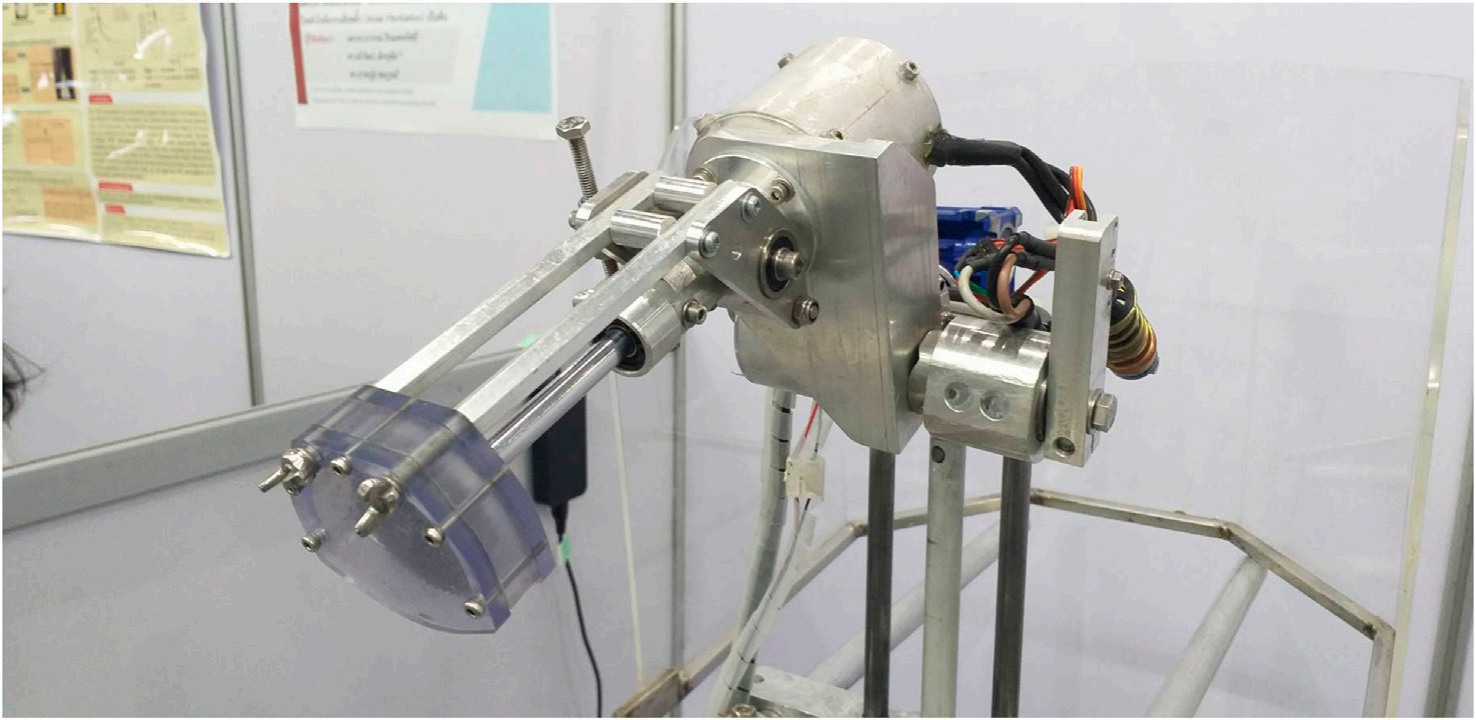
Closed-up view of the circular saw blade set covered by an acrylic protector for human safety and prevent secretion spreading.

As part of mechanical design, this robot machine consists of four parts. Firstly, the rotary direction set, for controlling the speed of the saw frame, from 0 to 1 RPM. Secondly, the circular saw blade set, for adjusting the speed of the saw blade from 0 to 6,300 RPM. Thirdly, the saw blade depth, which is controlled from 0 to 20 mm. Finally, the electrical circuit is attached to a battery, which works by an analog signal with Pulse width modulation (PWM) for stabilizing the signal. The remote control system is housed in the high-grade aluminum electrical control box, which consists of a digital battery voltage indicator with an LCD display, the buttons for controlling the speed of the saw blade, the depth of the saw blade, the rotation of the saw frame, and also the emergency STOP push button. The autopsy operator can stand away from the robotic autopsy saw and regulate it, from a radius of 2 m or further, depending on the cable length. The robotic autopsy saw was designed and tested with plastic skull models to ensure that it could cut the cadaveric skull perfectly (Jumlongkul and Chutivongse, 2019). The prototype of this machine is shown in Figures 2–4.

### Acoustic Measurement

All noise measurements were carried out during the skull cutting with a sound level meter (model TES-1351, resolution 0.1 dB, range from 35 to 130 dB, accuracy ±1.0 dB), which converts an acoustic wave to a unit of sound measurement (dB) (TES Electrical Electronic Corp, 2014). Two kinds of acoustic measurements were tested, including a peak sound level and a time-weighted average (TWA) over 30 s for each cadaver. These were analyzed by comparison of two cadaveric groups with an arithmetic mean ± SD using Microsoft Excel Office 365.

The continuous steady or fluctuating noise over 1 h was calculated from the equivalent noise level in 1 h (LAeq, Tr), calculated as follows:

LAeq, Tr = LAeq, Tm + 10 Log10 (TmTr)

Where Tr is the average noise level (60 min), Tm is the actual time (0.5 min), and LAeq Tm is the specific noise over a representative period, which reflects variations from the specific source (dBA).

The continuous steady or fluctuating noise (LAeq, Ts), measured 5 times for each cadaveric group. was corrected using:

$$\text{LAeq, Ts = 10 Log 10 }\{\left(\frac{1}{\text{Tm}}\right)\sum^{\text{​}}Ti10^{0.1\text{LAeq},\text{Ti}}\}$$

Where Ti is the duration of time from the origin of a source at i (min). LAeq, Ts was corrected with the constant volume adjustment and noted as LAeq, Tm in formula 1, which would be investigated for LAeq, Tr.

In all the experiments, the noise dosimeter was calibrated daily before collecting sound levels for each sample (Belcham, 2015).

### Aerosol Measurement

The aerosol particles, present in the air during the experiments, were detected using a portable real-time dust monitoring device (CASELLA CEL Micro Dust Pro, measuring range 0.001 mg/m^3^ to 250 g/m^3^) which samples total, respirable, PM2.5 or PM10 with an optional adaptor. This dust collector machine can detect the aerosol particles using a forward light scattering method which converts the signal to a unit of dust per volume (mg/m^3^) (CASELLA CEL Inc, 2012). Peak dust concentration levels and mean levels of dust concentration, over 30 s, during the experiments were collected. The data from each group was calculated and interpreted using an arithmetic mean ± SD by Microsoft Excel Office 365.

## Results

All the experiments were conducted by the same mortuary technician. From a total of ten cadaveric skulls, five were cut by the oscillating saw and five by the robotic apparatus. The auditory and dust measurements were as follows;

### Acoustic Profiles

The average noise levels in an autopsy room, during usual operations without machine saws working, were measured over 30 s at 71.7 dBA (data not shown in Table 1). The average noise levels during every experiment (LAeq, Tr) in the robotic autopsy saw group, measuring 58.9 dBA, was lower than the oscillating saw group, measuring 67.5 dBA, as well as Total LAeq, Tr (70.9 dBA with robotic autopsy saw and 75.5 dBA with oscillating saw, respectively). It should be noted that the total noise level (Total LAeq, Tr) for the robotic system did not exceed that of the usual noise level conducted without an electric saw. During parts of both of the saw procedures, the noise levels were low. However, the highest peak sound level was 99.7 dBA, which occurred during operation of the oscillating saw while the lowest peak was reported during the robotic procedure, measured at 81.0 dBA. The peak sound levels were in excess of 90 dBA in both groups. The calculated data were shown in Table 1.

**TABLE 1:** The noise exposures in an operating room during skull cutting. Measurements of the noise levels (Peak Level and LAeq, Tr) for each experiment, and the average noise level (Total LAeq, Tr) of the oscillating saw as well as the robotic autopsy saw.

Procedure	Average peak level (Min-Max) (dBA)	LAeq, tr in each cadaver (dBA)				Total LAeq, tr (dBA)
		Min	Max	Mean	SD	
Oscillating saw	94.0 (88.1–99.7)	64.6	71.4	67.5	±3.2	75.5
Robotic autopsy saw	86.9 (81.0–90.7)	44.2	67.6	58.9	±9.7	70.9

### Aerosol Particles

From Table 2, before the skull cutting experiments of two cadaveric groups, the average peak concentration of dust in the autopsy room, detected over 30 s, was 1.853 mg/m^3^ (the minimum value of 0.018 mg/m^3^ and the maximum value of 2.750 mg/m^3^, respectively) which was the greatest value when compared with the oscillating saw and robotic autopsy saw. The average peak aerosol concentration of the oscillating saw, measured at 0.431 mg/m^3^, was higher than the robotic machine procedure (0.346 mg/m^3^). Importantly, the average aerosol concentration before skull cutting was 0.153 mg/m^3^ (the minimum value of 0.016 mg/m^3^ and the maximum value of 0.299 mg/m^3^, respectively) which was the highest value among both of oscillating saw and robotic autopsy techniques. Nevertheless, the average aerosol concentration of the oscillating saw procedure, approximately 0.030 mg/m^3^ (the minimum value of 0.014 mg/m^3^ and the maximum value of 0.056 mg/m^3^, respectively), was at a lower level than the dust generated by the robotic method, measuring 0.106 mg/m^3^ (the minimum value of 0.016 mg/m^3^ and the maximum value of 0.148 mg/m^3^, respectively.

**TABLE 2:** Dust concentrations before skull cutting and during two different skull cutting procedures: oscillating saw and robotic autopsy saw. The peak concentrations, mean values, standard deviations, minimum and maximum levels of dust per volume over ten subjects.

Procedure	Peak concentration (mg/m^3^)		Dust per volume (mg/m^3^)	
	Mean (Min-Max)	SD	Mean (Min-Max)	SD
Before skull cutting	1.853 (0.018–2.750)	±1.276	0.153 (0.016–0.299)	±0.100
Oscillating saw	0.431 (0.116–0.706)	±0.221	0.030 (0.014–0.056)	±0.021
Robotic autopsy saw	0.346 (0.018–0.852)	±0.350	0.106 (0.016–0.148)	±0.052

## Discussion

Nowadays, the conventional procedure, which has been routinely applied for human skull cutting in forensic examinations, is an oscillating saw system. The robotic autopsy saw is recent development, using a robotic-assisted surgical technique. The first goal of this study, the measurement of noise pollution from the oscillating saw, was found to be greater than the robotic autopsy saw in all parameters. This might result from different techniques applied to the bone. The robotic saw blade system was designed based on a circular saw system which needs only a slow rotational speed of saw blade to cut the cadaveric skull while the oscillating saw uses a vibration method for breakthrough of solid objects. Therefore, the machinery strokes of the oscillating saw must be over 10,000/min, inevitably causing high noise generation (SCHREIBER GmbH, 2012). As suggested by the National Institute for Occupational Safety and Health (NIOSH) and the Occupational Safety and Health Administration (OSHA), total LAeq, Tr in both groups and the average noise level without saws working should not exceed the recommended exposure limits (REL) for noise, over an 8-h average (85 dBA suggested by NIOSH and 90 dBA by OSHA, respectively) and noise over a 15-min average (100 dBA suggested by NIOSH and 115 dBA by OSHA, respectively) while the skull cutting operations of this study did not exceed 3 min/test (Centers for Disease Control and Prevention, 2018).

In addition, the result of inhaled particle measurement in this study deviated from the hypothesis which we had proposed, that the robotic autopsy saw might reduce the rate of aerosol generation. Although the autopsy robotic machine worked with the dust collector covering the saw blade and the acrylic box covering a cadaveric head, which was designed to confine the bone dust particles, it still generated a peak dust concentration and dust per volume greater than the oscillating saw. Despite the results contrasted with the presumption before testing, the aerosol pollution, which came from skull cutting by the robotic autopsy machine, as well as the oscillating saw, were not greater than the dust concentrations before skull cutting. The described skull cutting procedures do not generate greater amounts of bone dust compared with the usual autopsy condition. The portable real-time dust monitoring device was 1 m away from the operation site, as is standard operating procedure, therefore, the bone dust might have had too far to diffuse to get into the probe chamber and so could not be adequately detected by the modulated laser light source.

As regards to the second goal of this study, trialing the replacement of the conventional oscillating saw with the robotic-assisted surgical technique, using the robotic autopsy saw. A new skull opening procedure to address concerns about the health and safety of forensic pathologists and their colleagues. This robotic machine works on a basis of remote control and electronic systems so the co-workers must be trained before using this application. The prototype of the robotic autopsy saw is very large when compared to the oscillating saw. The robotic autopsy machine should be modified into a much more compact size, linked with either a mobile phone application or other wireless application, and have a wider application which is not limited only for skull opening. To solve these problems, the cadaveric skull and tissue cutting manipulator was designed, fabricated, and preliminarily tested. This robot should be used as a comparative machine for the next experiment (Jumlongkul, 2020).

Future studies should attempt to evaluate the relevance of short-term and long-term effects, related to health problems and the type of machines used in an autopsy room, for example; occupational lung diseases, musculoskeletal disorders, occupational psychosis, and occupational infectious diseases in the workplace. The evidence of disease development in forensic healthcare workers must be considered and we will consider any idea, using robotic technology, to raise the standard of healthcare worker safety.

## Conclusion

In conclusion, this research study examined the effectiveness of a new method to cut the cadaveric skull, using robotic-assisted surgery, which resulted in decreased noise levels when compared with the oscillating saw method. This robotic machine, which is an optimal machine for post-mortem examination, also limited the release of aerosol particles to a level similar to common autopsy procedures not involving the use of operation of calvarial cutting tools. Nevertheless, further study is necessary to improve the appropriate autopsy instruments and we hope that this research will inspire many researchers to further consider the safety of health care workers.

## Data Availability

The original contributions presented in the study are included in the article/Supplementary Material, further inquiries can be directed to the corresponding author.
